# ‘The More We Stand For – The More We Fight For’: Compatibility and Legitimacy in the Effects of Multiple Social Identities

**DOI:** 10.3389/fpsyg.2017.00642

**Published:** 2017-04-26

**Authors:** Maria Chayinska, Anca Minescu, Craig McGarty

**Affiliations:** ^1^Department of Psychology, University of Milano-BicoccaMilan, Italy; ^2^Department of Psychology, University of LimerickLimerick, Ireland; ^3^School of Social Sciences and Psychology, Western Sydney University, SydneyNSW, Australia

**Keywords:** multiple social identities, perceived compatibility, perceived legitimacy of protest, collective action, political activism

## Abstract

This paper explores the expression of multiple social identities through coordinated collective action. We propose that perceived compatibility between potentially contrasting identities and perceived legitimacy of protest serve as catalysts for collective action. The present paper maps the context of the “Euromaidan” anti-regime protests in Ukraine and reports data (*N* = 996) collected through an online survey following legislation to ban protests (March–May, 2014). We measured participants’ identification with three different groups (the Ukrainian nation, the online protest community, and the street movement), perception of compatibility between online protest and the street movement, perception of the legitimacy of protest, and intentions to take persuasive and confrontational collective action. We found evidence that the more social groups people “stood for,” the more they “fought” for their cause and that identifications predicted both forms of collective action to the degree that people saw the protest and the online movement as compatible with each other and believed protest to be legitimate. Collective action can be interpreted as the congruent expression of multiple identities that are rendered ideologically compatible both in online settings and on the street.

## Introduction

A very relevant issue to address when examining the dynamics of grass-roots collective action is what type of commitments drive individuals’ behavior and how the real-world structural context conditions collective efforts to attain social change. Although collective action is routinely understood as efforts by members of a disadvantaged social group to overturn an injustice, the concept itself suggests the need to look beyond a single, nominal social category membership as the seed of dissent toward contested and multifaceted political agency and, thus, multiple politicized collective identities as a potential explanation of the drivers of social movements for social change. Did the Russian revolution of 1917 establish the dictatorship of the proletariat? Are efforts 100 years later to “make America great again” directed to the benefit of all (US) Americans? Perhaps, but it also seems plausible that in these, and many other cases, that there is a number of salient social categories that may be relevant at the same time for either a community or the same individual actor. The multiplicity of actors, political agendas and group identities are likely to achieve higher mobilization power in certain contexts, and social psychological models of collective action should be able to account for effects of such multiple identities.

The present research is designed to answer specific questions about how identification with distinct social groups coheres to underpin engagement in coordinated collective action. In particular, we aim to understand the process through which multiple social identities of self are translated into synchronized political action as well as conditions under which people opt for different forms of collective action. We seek to understand this phenomenon in relation to the wave of political activism in Ukraine starting in 2014.

Psychological research (as highlighted in this Topic) has demonstrated that people belong to a number of social groups and affiliations that can be potentially mobilized and politicized and has posited the question of whether these multiple commitments of self can lead to a synchronized expression (e.g., Cruwys et al., 2016; Curtin et al., 2016). Despite the increasing interest the underlying mechanisms of the expression of multiple identities remain unspecified.

The matter is complicated further because social movements may reflect not only multiple agendas but multiple methods. Some of these methods involve building support by persuading potential supporters to join a movement whereas others involve disrupting or even destroying opposition. Scholars have sought to understand the causes of extreme, non-normative and violent collective action (e.g., Moskalenko and McCauley, 2009; Thomas and Louis, 2014; Thomas et al., 2014; Becker and Tausch, 2015; Jiménez-Moya et al., 2015; Shuman et al., 2016) by distinguishing them from moderate, normative, and peaceful action. We appreciate that all of these distinctions have merit for various purposes In this study, we rely on the distinction between *persuasive action –*as a form of protest with the primary purpose of influencing/persuading third parties (or even opponents) to share a political goal and *confrontational action*, conceived as a form of protest that confronts opponents with direct action that may disrupt their activities. The advantage of this distinction is that labels for action such as “non-normative,” “unlawful,” “violent,” and “extreme” are subject to locally applicable definitions that are often within the power of authorities to define. This is generally problematic where those authorities are themselves the targets of action, but is specifically problematic where forms of protests are outlawed during the course of a campaign. In Tunisia in early 2010 street protests were both illegal and very uncommon (McGarty et al., 2014). Protests continued to be illegal right through to the point that the Ben Ali regime was overthrown but they had become common right across the country by early January 2011. In Ukraine in 2013/14 public demonstrations, most famously in the Maidan Square in Kyiv, had become regular and heavily supported events, but in January of 2014 they were declared to be illegal, prompting a new wave of intensified protests.

A recognition of context in promoting and constraining the expression of social identities has prompted analysis of the perception of political opportunities in relation to the anticipated outcomes of protest efforts (e.g., Williams, 2004; Reicher and Haslam, 2013; van Stekelenburg and Klandermans, 2013). However, little attention has been paid to the role of the perception of legitimacy of protest in predicting different forms of collective action.

The present research readdresses these issues and suggests a framework for understanding the expression of multiple social identities situated in a specific historical context. The key objective of the present research is, therefore, to examine the mechanisms behind a synchronized expression of multiple social identities in explaining persuasive and confrontational collective action. In line with the social identity approach, we first propose that collective action can be explained to a greater extent by accounting for multiple social identities whose ideological contents are aligned rather than by focusing on a singular salient category membership (hence the title of this paper ‘the more we stand for – the more we fight for’). In particular, based on self-expansion theory (Aron and Aron, 1996; Aron et al., 2004), we assume that people expand their self-concepts to include different identities of groups and communities they belong to, and this can occur without individuals necessarily incorporating or nesting one social identity into another. This psychological process, also referred to as the inclusion of other in the self, is thought to be achieved through an increasing overlap between the representations of self and social groups (e.g., Tropp and Wright, 2001).

Secondly, we argue that the ideological content of these identities need to be (or become) compatible with each other in order for them to drive collective action (see Bliuc et al., 2012; McGarty et al., 2014). We thus suggest that holding a shared (civic) vision based on the *perceived compatibility of multiple identities* provides solid psychological ground for engaging in collective action.

Another important consideration, in addition to the compatibility between identifications, is the degree to which the *political opportunity structure* (e.g., Tarrow, 1998; Meyer, 2004), that is, system-level constraints of individual-level intentions to take collective action, imposes a particular set of expectations regarding the ways in which those multiple identities may be expressed. In other words, if the norm that protest is a legitimate way of engaging in collective action is aligned with multiple identities, then the perception of protest as legitimate will help explain the effects of these identities on collective action. This is a particularly timely and contextually relevant operationalization, capturing people’s perception of a key feature of the political opportunity structure in contested times of transition.

### Compatibility of Multiple Identities and Political Activism

The idea that collective action may be explained through politicization of multiple social identities has recently received more attention in collective action research (e.g., Curtin and McGarty, 2016; Curtin et al., 2016; Louis et al., 2016; building on earlier insights by Klandermans et al., 2008; Simon and Ruhs, 2008).

It has been argued that the psychological processes behind the simultaneous expression of multiple identities might involve the formation of opinion-based groups (see McGarty et al., 2009), where the content of commitments (*what ‘we’ stand for and what ‘we’ stand against*, see Chayinska et al., in press), rather than strength and salience of social identification, has been shown to be the key factor to understanding politicization and action engagement. Smith et al. (2015b; see also Smith et al., 2015a) conceptualize this as the formation of an *identity-norm nexus* where people come to see shared views about how to change the world as an aspect of self. Qualitative analysis by Curtin et al. (2016) also revealed that individuals who experience marginalization and privilege *at the same time*, and arguably identify with advantaged *and* disadvantaged groups, tend to simultaneously express these multiple identities to the extent to which these identities may be subsumed under a broader identity category (i.e., interpretable as involving commitment to a common cause).

Turner-Zwinkels et al. (2015) have shown that politicization of social/personal identities is not merely a matter of increasing allegiance to multiple political agendas; it is the overlap in the normative content of these identities and a subjective internalization of their agendas through which the political becomes personal that predicts commitment and action. Similarly, Louis et al. (2016) contend that one of the reasons why activism in one domain (i.e., identification with Cause 1) might predict and facilitate the likelihood of activism in other domain (i.e., identification with Cause 2) is the ideological or normative alignment between these movements. According to these authors, it is therefore necessary to explicitly measure whether and how such a normative consensus leads to collective action.

Other scholars have highlighted that a meaningful interconnectedness of available multiple identities (e.g., Case et al., 2012; Greenwood, 2012) or so called ‘identity-value fit’ (e.g., Kutlaca et al., 2016) tends to facilitate their simultaneous expression, and that holding a number of social commitments, as opposed to a sparse social identity profile, is beneficial to life transitions. However, there appears to be one crucial condition: the multiple identities one holds need to be perceived to be compatible with each other (e.g., Benet-Martinez and Haritatos, 2005; Riketta and Nienaber, 2007; Iyer et al., 2009).

Taken together, these findings suggest that the more people perceive their multiple social identities to be compatible the higher the level of identity integration. Conversely, the perception of two or more identities being in opposition to each other, perhaps due to conflicting values and norms, signals a lower level of identity integration. We extend this line of research by suggesting that that expression of multiple politicized identities through collective action is more likely to occur when individuals do not have to make an ‘either–or’ choice between two or more commitments. In other words, when a high level of identity integration between multiple identities is present, thus when identities are perceived as more compatible, collective action is more likely to emerge.

Based on this literature review, we suggest that the greater the degree of normative or ideological compatibility between multiple social identities the more likely it is that they will lead to coordinated collective action for the same cause. Thus, the present study investigates the potential mediating role of perceived identity compatibility in the relationships between the identification with the online protest community and street movement and intentions to take persuasive and confrontational collective action. We conceptualize perceived compatibility between multiple identities as the extent to which their content (and the values assigned to it) are perceived at the individual level to be coherent and in congenial combination with one another. In other words, for multiple identities to be psychologically compatible, we assume, the identification with one social group must not be perceived as conflicting with identification with another group.

While we see ideologies as a perfectly viable basis for the formation of social identities (most obviously in relation to political groupings such as socialist and fascist) our focus here on the link between ideology and identity is chiefly in terms of the *perceived compatibility of identities* as they relate to participation in protest. Thus one pro-democracy, pro-European protester may hold an ideological commitment to non-violence or to obeying national laws (even when they are seen to be unjust) and another might believe that democratic ends justify violent means, or that ‘bad’ laws need to be broken. We turn to these matters now.

### Perceived Legitimacy of Protest

It has been widely accepted that the context within which politicized collective identities emerge plays an important role in the understanding of political collective action and its consequences. Past research has paid insufficient attention to the fact that the legitimacy of engaging in protest against authorities or for a particular cause is itself a very contested aspect of social structure, and therefore varies across political contexts. Although it is commonly taken for granted in liberal democratic settings that political structures accommodate the right for participation in protest, this is not true in most parts of the world throughout history. This pattern may potentially challenge the cross-cultural applicability of findings from Western democratic contexts to other contexts where transition between political regimes and democratization is an ongoing process and challenging reality.

Some political science research (e.g., Tarrow, 1998; Meyer, 2004; Corcoran et al., 2011) indicates significant links between democratization and protest such that a change in some dimensions of the political opportunity structure tends to affect an individual perception of the feasibility of protest. For instance, analyzing the data from the World Values Survey, Corcoran et al. (2011) have revealed that the perception of political institutions as open (a macro-level factor) affected individuals’ sense of efficacy (a micro-level factor), which in turn was found to determine intentions to take collective action. Social psychological research illustrated these processes in the analysis of McGarty et al. (2014) looking at the protests against repressive regimes in North Africa in 2010 and 2011. In this context, protest came to be seen as feasible after striking novel images of anti-regime protest were recorded on camera phones, uploaded to social media video sharing sites (e.g., YouTube), and from there, broadcast through external satellite television networks (Al Jazeera) to citizens in Tunisia and Egypt. Arguably in this context online mobilization was not alienated from street protest but was a precondition for it: part of a broader global pattern that Castells (2012) describes as the occupation of specific online spaces preceding the occupation of physical public spaces. Also, McGarty et al. (2014),’ research captures the transition in people’s perception that protest is “allowed,” and the agency with which actors expanded this legitimacy of protest from the online to the street contexts.

Perceiving protest to be a legitimate political act is likely to result in collective action involving conventional, persuasive forms of action, but perhaps less so in the more confrontational forms of action (e.g., Simon et al., 1998; Thomas and Louis, 2014; Becker and Tausch, 2015). In the present study we examine whether *perception of protest* as a legitimate instrumental tool to achieve social change may also be rooted in the process that governs expression of multiple politicized identities. Specifically, we suggest that the ideological alignment (i.e., perceived compatibility) between different social identities *along with* perceiving protest as legitimate will explain how the relevant politicized identities will generate collective action. These processes are assumed to explain why and how multiple identities may align to predict engagement in collective action. In other words: ‘the more we stand for’ (multiple identities), ‘the more we fight for’ (increased collective action), because ideologically ‘we’ are fighting for the same goals (perceived compatibility) and because ‘we’ perceive our actions in protest as legitimate (perceived legitimacy of protest).

### Current Study

We tested these ideas in the context of the 2014 Euromaidan movement – an uprising against the refusal of the then Ukrainian national government to sign the Association Agreement with the European Union. After a set of pro-Western protests – the Russian-aligned majority of the Parliament of Ukraine passed a set of anti-protest laws that included measures limiting street assemblies and internet freedoms (Cohen, 2014). The new laws, criminalized all unauthorized meetings and gatherings in public places, and the online dissemination of “extremist information” (without providing a clear definition of ‘extremist,’ Centre for Civil Liberties, 2014). In the space of a few months, the political opportunity structure changed: the legitimacy of protests came to be contested in the midst of a political identity crisis of allegiances toward Ukraine, Europe or the Russian-led Customs Union among protesting Ukrainians. This is an especially intriguing context because of the legislative change. A growing social movement that sought to promote closer ties with Western Europe was confronted with new laws that made both street and online protest illegal. Obviously, however, street protests remained more detectable and punishable by authorities and 82 of street protesters were killed, more than 1,100 injured and 234 arrested in the period after the new laws were introduced (Ukraine Crisis: Timeline, 2014).

We captured this moment in this study, looking at whether and how participation in an online protest movement become an acceptable alternative to street protest, whether online activism may represent the legitimate continuation of the protest by other means in order to preserve the future of Ukraine, or whether online protest become an unsatisfactory and alienated substitute: expressing what Morozov (2009, 2011), Gladwell (2010) and others might deride as slacktivism, clicktivism or even in Morozov’s terms “the net delusion” (see Schumann and Klein, 2015; Thomas et al., 2015).

We tested a model in which perceived identity compatibility and perceived legitimacy of protest mediate the relationships between multiple identities and collective action. We expected that, in the context of anti-government protest, people may find that there are more than one group or community that best represents their interests, and if they perceive that the values of these several groups are compatible (not conflicting), they will be likely to express their joint claims on behalf of those communities (the more we stand for – the more we find for).

We included three different social identifications as predictors of action: identification with the street protest movement, identification with the online protest movement, and Ukrainian national identification. We expected all three to be relevant predictors but the inclusion of national identification allowed us to address the possibility that identification with the single most relevant existing social category could provide an adequate (and parsimonious) account. Ethnic identification in terms of Ukrainian and Russian heritage represented other alternatives to measure single identities, and may seem obvious choices to external observers in view of recent dramatic conflicts in Ukraine. However, the civic ideology of the modern Ukrainian state (in which most participants would have been socialized) eschewed categorizations based on ethnicity in favor of a wider national identity category (see Prizel, 1998).

We expected that both persuasive and confrontational forms of collective action would flow from identification with the three different social identities (identification with Ukraine, identification with the online protest community, and identification with the Euromaidan street movement). Moreover, perceived compatibility and perceived legitimacy are expected to explain the effects of multiple identities on collective action. We generally also expected that the predictive power of the model including multiple identities and perceived compatibility and legitimacy will be stronger for persuasive than confrontational forms of collective action. This is because when considering persuasive collective actions, people are more likely to act out of a coherent ideological alignment between their multiple identities and the normative beliefs about these identities and about protest. When it comes to confrontational forms of collective action, this alignment between identities and normative beliefs might not be necessary. We tested these hypotheses with survey data collected during the 2014 protests.

## Materials and Methods

### Participants and Procedure

Participants were approached through a public online survey posted to Facebook pages that were generally discussing political events in Ukraine. The data were collected between March 28 and April 30, 2014, (as soon as possible after the January 26 passage of laws that restricted people’s right to protest led to larger protests in Ukraine). The questions of the survey focused on socio-demographics and attitudes toward current political issues. The items were available in separate Ukrainian and Russian versions of the survey instrument. In order to guarantee coherence and validity of the questions, all items were translated from English to Ukrainian/Russian and back using a standard translation-back-translation procedure (Brislin, 1970). Participants were required to be of Ukrainian nationality and aged over 18.

In total, the responses from 996 participants were used in the data analysis. The sample ranged in age from 18 to 77 (*M*_age_ 33.87 years, *SD* = 9.61) and comprised 51.7% women. Participants were highly educated (57.4% having graduated from university), 44.2% were employed full time, and 57% indicated Ukrainian as their first language. Some 72.7% reported that they completed this survey while in Ukraine, 24.7% – while living abroad (mostly in European countries, 15.5%, and in North America, 4.4%).

### Measures

#### Socio-Demographics

Participants indicated age, gender, ethnicity, country of birth, current residence, prior experience of living abroad, educational level, employment status, and mother tongue (i.e., Ukrainian, Russian, other).

#### Identification with Online Protest Community and the Street Movement

We measured self-expansion with the online protest community and with the street movement using a modified Inclusion-of-the-Other in-the-Self-Scale (the IOS-scale, Aron et al., 1992). The IOS task depicted five pairs of circles (numbered one to five), ordered by degrees of increasing overlap between the pairs. Self-expansion refers to a “fundamental human motivation to enhance potential self-efficacy (which is the ability to accomplish desired goals by attaining) greater material, social, and informational resources” (Aron and Aron, 1996; Aron et al., 2004). Participants were asked to indicate how close they felt toward online protest community and street movement, respectively, by selecting one of the five pairs of circles. Higher numbers are indicative of a smaller felt distance between oneself and others participating in the movement.

#### Identification with Ukraine

Six items from Leach et al. (2008) were used to measure identification with Ukraine. These and other measures below used five point Likert scales labeled from 1 (Strongly disagree) to 5 (Strongly agree). These items captured Leach et al. (2008) dimensions of centrality (e.g., “I often think about the fact that I am a part of the Ukrainian people”), satisfaction (e.g., “I am glad to be part of Ukraine”), and solidarity (e.g., “I feel solidarity with people in Ukraine”) of identity, that comprise the second order dimension of group-level self-investment and are considered to be particularly important for collective political action. The items were averaged to form a composite measure of identification with Ukraine [Cronbach’s alpha (α) = 0.95].

#### Perceived Compatibility

To measure perceived compatibility between the online protest community and the street movement we used four items adapted and modified from Riketta and Nienaber (2007): ‘this online community is another platform for the street protest,’ ‘by becoming members of Online Protest Community people safeguard the very existence of the street protest,’ ‘in general, the mission statement of Online Protest Community fits well with the mission statement of the street protest,’ and ‘the ideas of Online Protest Community concerning interaction and cooperation correspond to the ideas of the street protest’), α = 0.79.

#### Perceived Legitimacy of Protest

Beliefs about legitimacy of protest were assessed using a 6 item scale: ‘These people were wasting their time protesting (recoded),’ ‘I think protesting on the streets was a valid form of behavior in Ukraine,’ ‘Protesting changed nothing (recoded),’ ‘I think this was irresponsible behavior (recoded),’ ‘I think there should be more protests in Ukraine,’ ‘This was not typical Ukrainian behavior,’ α = 0.71.

#### Persuasive and Confrontational Collective Action

Respondents were asked to indicate how willing they were to participate in 10 different offline collective actions. Principal components analysis yielded two components with eigenvalues greater than 1 that accounted for 55.45% of the variance. Loadings, after oblique rotation, revealed that relatively non-violent, persuasive actions (e.g., ‘voice group’s claims in social network pages,’ ‘display symbolic attributes (flags, stripes) of my group, ‘participate in marches and motorcades,’ ‘donate money for the cause of my group,’ ‘compile a blacklist (list for lustration, sanctions),’ and ‘participate in flash-mobs and art events organized to support the cause of your group’) loaded primarily on the first component (41.10%); seemingly extremely confrontational actions (e.g., ‘blockade activity of ideological opponents,’ ‘sneer at opponents’ symbolic attributes (e.g., flags),’ ‘participate in mock political funerals,’ ‘sabotage political events of opponents’) loaded on the second component (14.35%). The items were averaged to yield composites of individual’s likelihood to engage in persuasive (α = 0.84) and extremely confrontational (α = 0.70) collective action. The two scales were moderately correlated (*r* = 0.592, *p* < 0.001).

## Results

### Statistical Analyses

The preliminary analyses involved bivariate analysis and hierarchical multiple regression. In this step, predictor variables were centered when computing interaction terms to minimize colinearity. The main analysis involved a test of the mediational model. Data analyses were performed using SPSS 24 and Amos 24. Fit statistics, including χ^2^ test (which can be affected by sample size), root mean square error of approximation (*RMSEA*), comparative fit index (*CFI*), and Akaike Information Criterion (*AIC*) were evaluated (Kline, 2011). The standardized paths between the variables included in the model were examined. The magnitude of effect sizes for the regression paths was determined as 0.10, 0.30, and 0.50 for small, medium, and large effects (Cohen, 1992). A *p*-value of less than 0.05 was considered to be statistically significant in all of the analyses.

### Preliminary Analysis: Do Multiple Politicized Identities Predict Collective Action?

Data screening was performed to ensure there were no violations of the assumptions. The descriptive statistics and bivariate correlations are presented in **Table 1**.

**Table 1:** Correlations and descriptive statistics for all variables (*N* = 996).

	Variables	1	2	3	4	5	6	7	*M*	*SD*
1	Identification with Ukraine	–	0.274	0.263	0.317	0.475	0.402	0.179	4.49	0.96
2	Identification with OPC		–	0.396	0.383	0.359	0.467	0.306	3.26	1.14
3	Identification with SM			–	0.216	0.357	0.524	0.297	2.84	1.13
4	Perceived compatibility between OPC and SM				–	0.406	0.425	0.228	3.77	0.87
5	Perceived legitimacy of protest					–	0.550	0.287	4.33	0.76
6	Persuasive collective action						–	0.592	3.73	0.89
7	Confrontational collective action							–	2.74	1.04

*OPC, online protest community; SM, street movement, all correlations *p <* 0.001.*

First, we performed a hierarchical multiple regression analyses to test whether identification with several politicized categories predicts collective action better than one salient identity and whether interaction terms should be included in a final model along with main effects^1^. Overall, the regression analyses indicated that multiple identities have additive positive effects on both types of collective action (on persuasive collective action, *adjusted R*^2^ = 0.403, on confrontational: *adjusted R*^2^ = 0.132) and that adding perceived compatibility and perceived legitimacy of protest significantly improved the explanatory power of both models, respectively, *adjusted R*^2^ = 0.49, Δ*F*(2,994) = 89.41, *p* < 0.001, and *adjusted R*^2^ = 0.153. Δ*F*(2,994) = 13.03. Details of the regression analyses are available from the corresponding author.

### Main Analyses: Do Compatibility and Legitimacy Mediate the Effects of Multiple Identities on Participants’ Intentions to Engage in Persuasive and Confrontational Collective Action?

**FIGURE 1:**
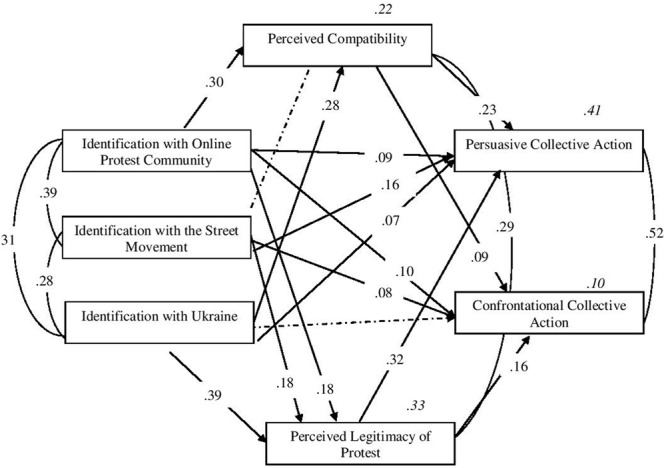
**Path analysis.** Model for the pathways to persuasive and confrontational collective action via perceived compatibility and perceived legitimacy of protest. Figure contains standardized parameter estimates, all *p* < 0.001. Non-significant paths are shown as broken arrows.

We tested a model in which perceived legitimacy and compatibility were considered as possible mediators of the effects of the three forms of identification on persuasive and confrontational action. Correlated error terms were allowed at each layer of the model. After the initial runs, we adjusted the models by setting two paths that had non-significant regression weights in the original models to zero, in particular the paths from the street movement identity to perceived compatibility (β = 0.03, *p* = 0.207) and from the Ukrainian identity to confrontational collective action (β = 0.01, *p* = 0.738). **Figure 1** shows the adjusted fitting model. The values for this final model fall within the cut-offs as advocated by Bentler and Bonett (1980) indicating good model’s fit: χ^2^(2) = 1.594, *p* = 0.451, *CFI* = 1.000, *RMSEA* = 0.000 (confidence interval: Low = 0.000, High = 0.043), *PCLOSE* = 0.978, *AIC* = 67.594.

The final model shows that identification with the street movement was a significant direct predictor of both persuasive and confrontational action and that identification with the online protest movement was a direct predictor of only persuasive action. Ukrainian national identification was an indirect predictor of both forms through perceived legitimacy and compatibility.

## Discussion

This study explored the mechanisms by which multiple identities predict collective action. First, we found empirical support to our hypothesis that collective action can be explained to a greater extent by accounting for multiple social identities with potentially aligned contents rather than by focusing on a singular salient category membership (‘the more we stand for – the more we fight for’). Importantly, these relationships were found to be significant for both persuasive and confrontational forms of collective action.

The effects of identification with the online protest community are noteworthy. In particular, we found that both persuasive and confrontational collective action were predicted by identification with the online protest community due to increased perceptions of compatibility between the online and the street protest, but also due to the higher perception that protest is legitimate. These findings are intriguing as they contribute to the ongoing research on political participation through the Internet (e.g., Schumann and Klein, 2015; Thomas et al., 2015), which has been criticized as a low-cost and low-risk activism lacking commitment and social impact (e.g., Morozov, 2009; Gladwell, 2010). Our results indicate an alignment of identification with different groups, irrespective of the online-offline divide, and perceived compatibility between identifications and the perceived legitimacy of protest seems to equally and independently predict collective action.

In our case there was no evidence that online protest was seen to be a defective or unsatisfactory form of action even alongside widely disseminated images of street protests that were globally distributed. It is important to bear in mind though that in Ukraine in 2014, as in other parts of the world, both online dissent and street protest were illegal. The Ukrainian government may have unwittingly increased the value of online dissent by banning it at the beginning of 2014, in the midst of the political crisis. Additionally, the online community of protestors offered a platform for the Ukrainian diaspora to become involved, whereas their participation in the street protests was logistically difficult if not entirely impossible. Future research may examine longitudinal changes in the relationships between multiple identities across various platforms of collective action, using individual-level analysis to track people’s enduring participation in fighting for a common cause.

Secondly, our data revealed that, beyond the direct effect of multiple identities, the perceived compatibility between them adds to our understanding of people’s engagement in collective action and explains the effects of identifications with the online community and with Ukraine. *What* “we stand for” and *how* we “stand for” the multiple communities we belong to (online, at the more abstract level of the national community), is of equal if not additional importance to our single memberships in any of them. Our study contributes to the theoretical discussion regarding the role of normative overlap between the agendas of different social groups in explaining cross-domain activism (e.g., Curtin et al., 2016; Louis et al., 2016) and long-term commitment to political causes (e.g., Smith et al., 2015b; Turner-Zwinkels et al., 2015). The results offer the interpretation that participation in collective action came to express national identification where Ukrainians saw protest as the right (legitimate) thing to do and where they perceived online and street protest to be compatible.

There was a seemingly paradoxical effect consistent with the perceived compatibility between street and online community identities mediating the effects of national identification on collective action but not the effects of street movement identification. In other words, the connection of the street Euromaidan movement with action was direct, so that supporters of the movement took to the streets. The connection of national identification with collective action, however, appeared to be indirect. In other words, the clear pathway between Ukrainian identification and collective action rested on perceiving the street movement and online movement were compatible with each other, because, we surmise that these two identities were themselves held to be valid expressions of Ukrainian identity (from the perspective of those supporters). We cannot know in retrospect whether that compatibility was a specific element that was relevant to the contested context of a nation that, at the time of data collection, was on the precipice of civil war or whether these patterns would obtain more broadly.

One possible way of thinking about these findings is that a capacity to form, synthesize, politicize and merge several opinion-based groups centered on short-/medium-term issues into a multi-goal campaign may serve as a key factor to understanding the processes behind coalition buildings and global activism. This also helps us understand the failures of mobilization: when networked campaigns use a vague idea-framing and related ideological noise that cannot justify involvement for a global cause, thus failing to bring people together. At the individual level, failure to cohere multiple group memberships into concerted collective action could be explained by exactly this lack of ideological overlap and miss-specification of the identity-norm nexus (Smith et al., 2015b). Although the present study was not designed to explicitly measure a link between formation of identities with overlapping injunctive contents and coordinated collective action, we believe that the curvilinear nature of this relationship requires a further examination.

Finally, consistent with our expectations, we found that expression of multiple social identities through collective action was also explained by individual perceptions of legitimacy of protest. Specifically, our findings indicate that higher degrees of identification with all politicized identities led to increased perceptions of protest as a legitimate method for achieving social change, and thus to higher likelihood of engaging in persuasive and confrontational collective action. In fact, our theoretical analysis helped us to identify and test this intriguing puzzle within the context of Ukraine, in the immediate aftermath of the introduction of criminal penalties for political dissent. The revealed pattern is important as it suggests that recognizing both between-group and inter-personal variations in people’s beliefs about protest (and incorporating the concept of perceived legitimacy of protest in collective action research) can help explain more general processes of choosing tactics from a spectrum of possibilities within a repertoire of contention. It is noteworthy to highlight that the effects of identifying with the street protest were only mediated by the perceptions of protest legitimacy and not by perceiving identity compatibility. Capturing people’s perceptions of protest legitimacy is also a way of operationalizing people’s engagement with the political opportunity structure, at times of political change and transition. This is much needed for developing a more dynamic theoretical model of the multiple links between identity and politics in constantly changing political environments.

Our findings raise other important questions: whether and under what conditions radicalization (confrontational political action) emerges from activism (non-violent political action)? To what extent do the tactics that one employs depend on political circumstances (e.g., legal criminalisation of dissent) and will variations in perceived legitimacy of protest produce similar patterns of collective behavior in both liberal and developing democracies? In other words, if variances in the perception of legitimacy of protest can help explain particular cases, can this conceptual approach generate testable models that hold across contexts? The answers are beyond the scope of this paper, but one factor may be due to the individual perception of political opportunities (e.g., Meyer, 2004) and, therefore, an elaborated conception of perceived legitimacy of protest that considers a broad range of conjunctural and issue-specific factors is recommended for future research.

To sum up, our findings support the idea that the expression of multiple politicized identities—their *agency*—can be understood to a greater extent when considering the political context and the rules of the game in which those identities are endorsed and internalized — that is, the surrounding ideological and political opportunity structure. However, it is important to advance our understanding of how various real or virtual communities, structured around non-contiguous spaces, may trigger confrontational (potentially radicalized) and persuasive collective behavior. Our models explained the latter to a greater extent, but not the former. Finally, we urge collective action research to continue to operationalize and test how the fluidity of the political opportunity structure affects the emergence of social and political identities, and the relationships of compatibility or opposition between these identities. A more complex framework capturing the diversity and multiplicity of identities (and relationships between them: such as perceived compatibility) as well as their relationship to the political context (the political background of legitimacy) will better equip us to understand and predict the paths to social change.

### Limitations

Reflecting on the external validity of our findings, we must exercise caution, due to the cross-sectional nature of our design, sample characteristics, and our use of self-report explicit measures of various politically sensitive issues. Therefore, although we obtained a large general community sample (in two languages), at a crucial time of the political crisis (shortly after the passing of laws that restricted people’s right to protest), we cannot account for the potential selection bias in the sample, or for the powerful effect of ‘history’ happening at the time of the study. While we do not wish to assume causal links between, for example, perceiving protest as legitimate and engaging in certain types of collective action, we would still like to argue that it is important to capture the variation in people’s beliefs about protest in a model predicting collective action. These variations will naturally be in tune with the changes in the political structure, and they are likely to have been particularly relevant for the Ukrainian setting. Further studies at different times in the development of a political crisis, and in contexts with variable degrees of democratization, will strengthen our empirical and theoretical ability to predict collective action.

Secondly, our results support the notion that the perceived compatibility between multiple identities is an independent predictor of collective action in addition to the combined effects of multiple politicized identities. This invites further refinements of the measures of compatibility and the three identification types. We assessed identification with three categories by using two different scales (i.e., item-based for national identification with Ukraine and the IOS pictorial measures for identifications with the online protest community and the street movement). This methodological discrepancy may account for the relative small covariance of these identifications. It is important to monitor how our understanding of the effects of multiple identities on collective action may depend on the measurement type. At the same time, we would note that concerns about ecological validity should prevail over the exclusive reliance on conventional measures. It might have been much more intuitively easy for people to respond with a pictorial measure when thinking about their self-inclusion in communities that were new and emerging at the time of the study. At the same time, when assessing national identification, the more established measures are perhaps best to assess the depth and strength of people’s group attachments.

Lastly, we operationalized perceived compatibility using several questions about the overlap between identity categories (i.e., referring to their ideological content). We did not explicitly measure the specific normative content and normative compatibility of the groups’ political agendas. This measure might seem, on one hand, comparatively superficial. On the other hand, its predictive validity indicates that participants responded to these questions with the two communities (online and street protest) in mind. Once more, due to the emerging nature of these group identities and communities of protest, in the midst of the political instability and crisis, a more in-depth measure might have been both impractical and unnecessarily complicated. This leaves room for future research to test whether normative compatibility between multiple politicized identities explains other intergroup behaviors, beyond predicting collective action.

## Conclusion

Summing up, we propose that collective action in the 2014 Euromaidan protests can be interpreted as the congruent expression of multiple identities that are rendered ideologically compatible both in online settings and on the street. This study investigated multiple identities that are related to the specific political context of a country in transition to democracy, caught in months-long upheavals and street protests, at a time when online interactions allowed for increased transnational mobilization and involvement in politics. The questions were: how do people negotiate their identities with their country, the online community of protesters and the street movement? Would these identities converge to support a concerted political agenda, thus increasing collective action intentions? Or would they be redundant in capturing people’s feelings and engagement with the various groups? In addition, how do these multiple identities relate to the political opportunity structure where protest itself was classified as illegal by the government, in a country grappling with an emergent democratic culture? We found evidence that the more social groups people “stood for,” the more they “fought” for their cause and that identifications predicted both forms of collective action to the degree that people saw the protest and the online movement as compatible with each other and believed protest to be legitimate.

We explained persuasive form of collective action to a greater extent compared to the confrontational form of action. Perhaps, negotiating multiple identities and looking for ideological alignment is a strategy that is more easily employed by those with moderate political agendas. Future research should explore how the dynamics between multiple identities (creating dissonance and lack of compatibility) might be employed to temper engagement in more confrontational or radical political actions.

## Ethics Statement

This study was carried out in accordance with the recommendations of Research Ethics Committee, University of Limerick with written informed consent from all subjects. All subjects gave written informed consent in accordance with the Declaration of Helsinki. The protocol was approved by the Research Ethics Committee.

## Author Contributions

This research is a part of a Ph.D. project of MC, developed under the supervision of AM in collaboration with CM. All authors contributed equally to the preparation of this manuscript.

## Conflict of Interest Statement

The authors declare that the research was conducted in the absence of any commercial or financial relationships that could be construed as a potential conflict of interest.

## Footnotes

1 The preliminary analysis includedcentred interaction terms between each of the two identities (i.e., identification with the online protest community and with the street movement), and perceived compatibility, as well as perceived legitimacy of protest. In fact the only significant interaction term was in the model explaining persuasive collective action where the interaction of the effect of identification with the online protest community and perceived compatibility was significant: β = 0.08, *p* = 0.037. For reasons of parsimony and in view of the high power derived from the large sample size we do not consider these interaction effects in the main analyses.
